# Dataset of physiological signals in the use of advanced driver assistance systems (ADAS)

**DOI:** 10.1016/j.dib.2026.112718

**Published:** 2026-03-26

**Authors:** Gabriel Martins de Castro, Rita de Cássia Silva, Alessandro B. de S. Oliveira, Cristiano Miosso

**Affiliations:** Faculty of Engineering Science and Technology, University of Brasilia Setor Leste Projeção A - Gama Leste, Brasília DF, 72444-240, Brazil

**Keywords:** Advanced driver assistance systems (ADAS), Physiological signals, Human–machine interaction, Driver monitoring, Signals dataset

## Abstract

This article introduces a dataset that investigates the physiological responses of drivers when using advanced driver assistance systems (ADAS) in real-world traffic conditions. The study, conducted in the Federal District, Brazil, involved seven drivers in controlled driving sessions. The time of day and the days of the week were standardized to ensure comparable traffic conditions. The data collection was centered on ADAS Level 2 systems, specifically the Lane Keeping Assist System (LKAS) and the Forward Collision Warning System (FCWS). The dataset includes five physiological signals: respiration, heart rate, galvanic skin response (GSR), leg muscle activity, and brain activity. These signals were continuously acquired using a dedicated instrumentation system installed in the vehicle. Given the complexity of collecting data under real traffic conditions, the acquisition sessions generated a large volume of raw data. Considerable post-processing was conducted to identify and segment portions of the signals with sufficient integrity for subsequent analysis. The dataset is structured as time-stamped raw signal spreadsheets, each corresponding to a specific driver and direction of the pre-established route (outbound and return). Such organization enables researchers to navigate the dataset easily, explore specific segments of interest, and conduct comparative analyses across participants and varying traffic conditions. The dataset is relevant to researchers in biomedical signal processing, driver state monitoring, intelligent transportation systems, and human–machine interaction. It may be used by academic laboratories investigating physiological responses during driving tasks, as well as by engineers and developers working on advanced driver assistance systems (ADAS), including automotive manufacturers and ADAS technology suppliers. The dataset, which includes synchronized physiological and vehicle dynamics data collected under real traffic conditions may contribute to the study of human responses during semi-automated driving, supporting research and development of driver-centered mobility technologies.

Specifications Table

**Table:** 

Subject	Engineering & Materials science
Specific subject area	Real-world acquisition of physiological signals during driving; Level 2 ADAS operation (LKAS and FCWS); Synchronized physiological and vehicle dynamics time-series; Focus on human responses in semi-automated driving.
Type of data	Time series saved in comma-separated-value (CSV) files.
Data collection	Physiological data were collected from seven drivers during real traffic sessions using ECG, EMG, EEG, GSR, and respiration sensors. Signals were acquired using AD8232, LM324, and T084 amplifiers, a NeuroSky MindWave Mobile 2 EEG (modified), a nasal cannula with a pressure transducer, and electrodes positioned according to biomedical standards. An NI USB-6009 board received all sensors, then signals were recorded via LabVIEW 2024. Vehicle data, in case speed and acceleration were simultaneously unregistered, using a Kingbolen OBD II ELM327 interface with Torque Pro.
Data source location	Institution: University of Brasília – Faculty of Science and Technologies in Engineering City: Gama East Country: Brazil
Data accessibility	Repository name: Dataset of physiological signals in the use of advanced driver assistance systems (ADAS) Data identification number: 10.17632/3s64m4mzsk.1 Direct URL to data: https://data.mendeley.com/datasets/3s64m4mzsk/1
Related research article	Castro, G. M., Silva, R. C., Mendes, C. J. M. R., & Oliveira, A. B. S. (2024). Physical and cognitive driver reactions characterization in response to active safety systems: A preliminary study. SIMEA 2024 – International Symposium on Automotive Engineering. SAE International. 10.4271/2024–36–0136.

## 1 Value of the Data

• This dataset provides synchronized recordings of five physiological signals (EEG, ECG, EMG, GSR, and respiration) and vehicle dynamics (speed and acceleration), acquired at 2 kHz under real-world traffic conditions in a vehicle (Jeep - Renegade, 2023), which is a Level 2 ADAS operation (LKAS and FCWS). The common sampling configuration and timestamp-based synchronization make easier joint analysis of physiological signals and vehicle dynamics in relation to driving events.
• The data are organized as raw time-stamped spreadsheets segmented by participant and by route direction, allowing structured comparison across drivers and driving segments. Such an organization supports realistic reuse scenarios, e.g., signal processing method, feature extraction, cross-signal correlation analysis, and exploratory driver-state modeling under naturalistic driving conditions.
• The dataset is relevant to researchers in biomedical signal processing, driver monitoring, intelligent transportation systems, and human–machine interaction. It may support academic laboratories in transportation engineering, research about the effect of human factors on driver behavior, and automotive systems research investigating physiological responses during semi-automated driving. Engineers and developers at automotive manufacturers, ADAS suppliers, and mobility technology companies may use the dataset to explore driver-state estimation approaches in Level 2 automation contexts.
• Multi-signal datasets collected in real traffic environments remain relatively scarce due to technical and safety constraints; this dataset may serve as a methodological reference resource for small-cohort naturalistic driving studies. Its documented acquisition protocol, standardized route, and consistent sampling parameters enable comparison with similar experimental setups.
• The dataset includes recordings from seven drivers and is not intended for population-level inference or statistical generalization. Nevertheless, its standardized acquisition protocol, synchronized multi-signal architecture, and real-traffic implementation provide a reproducible and methodologically transparent foundation for hypothesis generation, and comparative research on human responses during Level 2 ADAS operation.

## 2 Background

Advanced Driver Assistance Systems (ADAS) represent a key step toward safer and more automated mobility, yet their integration with human drivers remains complex. Earlier studies have highlighted that driving involves both cognitive and physiological responses, which can be captured through physiological signals such as heart rate [1], respiration [2], galvanic skin response [3], muscle activity [4], and brain signals [5]. These physiological indicators are known to vary with task difficulty, attention, decision-making, and emotional valence [6], making them valuable for examining human interaction with automation.

Despite growing research on ADAS, naturalistic datasets, combining vehicle dynamics and multiple physiological signals under real traffic conditions are still scarce, especially in the Brazilian context. Collecting such data is methodologically challenging due to signal noise, variability, and the constraints of real driving environments [7,8]. These operational limitations frequently restrict sample size in naturalistic multi-sensor studies, as the installation of biomedical sensors, real-time in-vehicle monitoring, and route standardization make large-scale acquisition difficult.

Existing physiological driving datasets often rely on simulators or controlled laboratory environments, which allow larger cohorts and detailed annotation but do not fully reproduce the variability and unpredictability of real traffic scenarios. Tao et al. [9] released a multimodal physiological dataset collected in a driving simulator to support driver behaviour analysis using synchronized EEG, ECG, GSR, and other biosignals, facilitating larger sample sizes. Meteier et al. [10] have explored drivers’ workload under conditionally automated driving using synchronized physiological signal acquisition in a controlled experimental setting. While such datasets provide valuable controlled resources, they typically do not combine on-road acquisition, continuous vehicle telemetry, and active Level 2 ADAS operation within a single synchronized framework.

In contrast, naturalistic data sets typically prioritize ecological validity and synchronized multi-signal acquisition, often at the expense of scale. Publicly available resources integrating synchronized physiological recordings with vehicle dynamics during real-traffic Level 2 ADAS operation remain limited. In this context, the present dataset contributes as a structured naturalistic resource that integrates multiple physiological signals with vehicle dynamics during Level 2 ADAS operation. It enables further investigation of human responses in semi-automated driving contexts. A preliminary methodological study previously investigated the feasibility of the instrumentation under controlled laboratory conditions, focusing on sensor validation and signal acquisition procedures. However, that study did not involve on-road data collection or structured dataset organization.

## 3 Data Description

The dataset is organized into folders and subfolders to provide easy access and reuse. Each folder corresponds to a specific driver and is subdivided by travel. Into each subfolder, raw physiological signals and vehicle dynamics data are provided as time-stamped spreadsheets. A summary of the dataset structure is presented in Table 1, showing the organization of drivers, routes, and data types.

**Table 1:** Dataset structure with folders, subfolders, and file types.

Folder	Subfolder	File type	Content description
/1- Driver 01/	/date Outbounb/	.csv	Raw signals (ECG, EEG, EMG, GSR, Respiration) + OBD data
	/date Return/	.csv	Raw signals (ECG, EEG, EMG, GSR, Respiration) + OBD data
/1- Driver 01	/OBD Outbound/	.csv	OBD data
	/OBD return	.csv	OBD data
/TrafficData/	—	.xlsx	Flow of vehicles by day and time from DER-DF
/Documentation/	—	.pdf	Acquisition protocol, sensor placement, LabVIEW diagram

The physiological data include five channels: electrocardiogram (ECG), electroencephalogram (EEG), electromyography (EMG), galvanic skin response (GSR), and respiration. Vehicle data, including speed and three-axis acceleration, was collected via the OBD II interface and exported as text files. Each acquisition file contains approximately 12,000 rows of time series data, with sequential file numbering used to preserve continuity during long driving sessions.

**Fig. 1:**
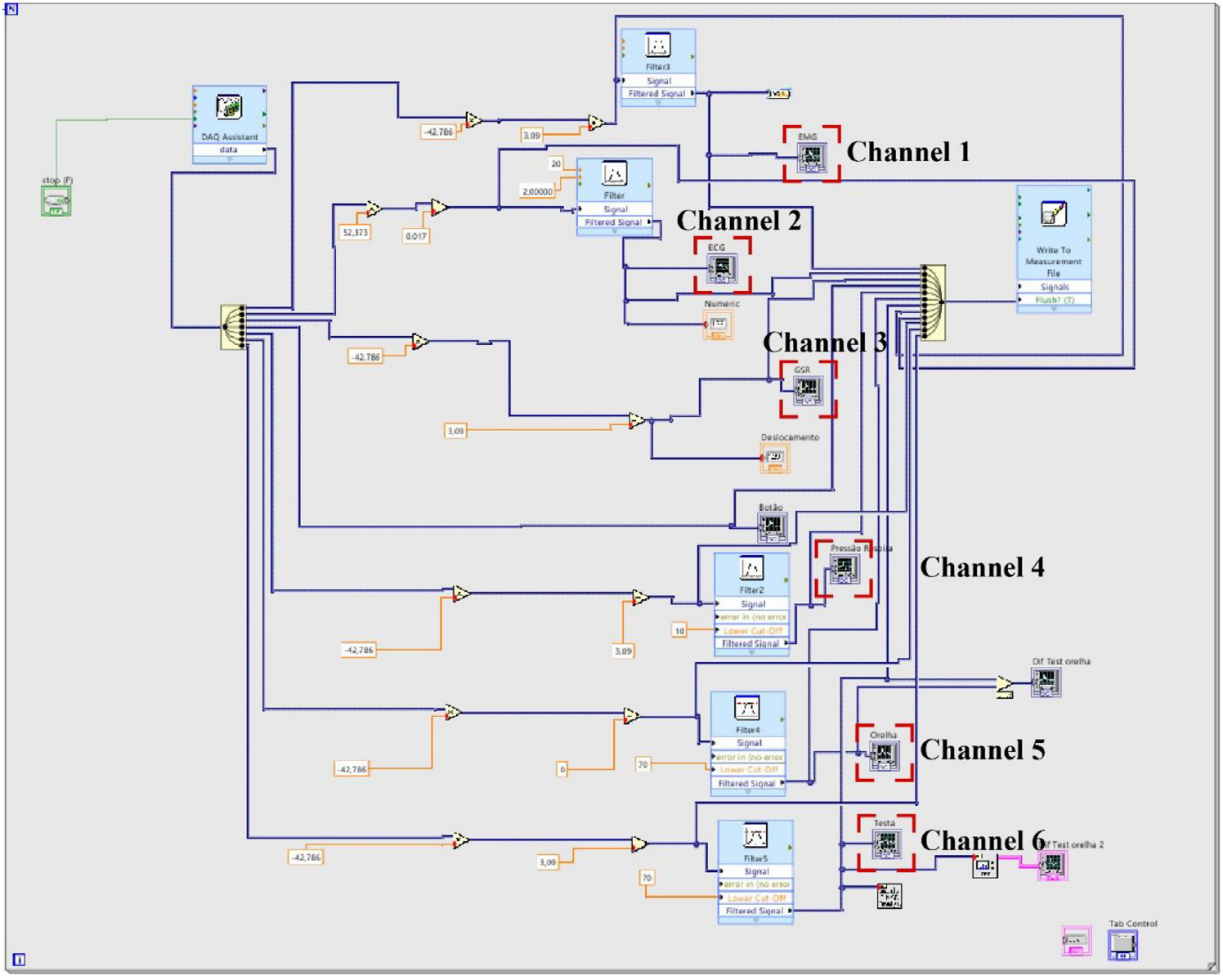
LabVIEW program developed for acquisition of physiological signals, representing the different acquisition channels.

**Fig. 2:**
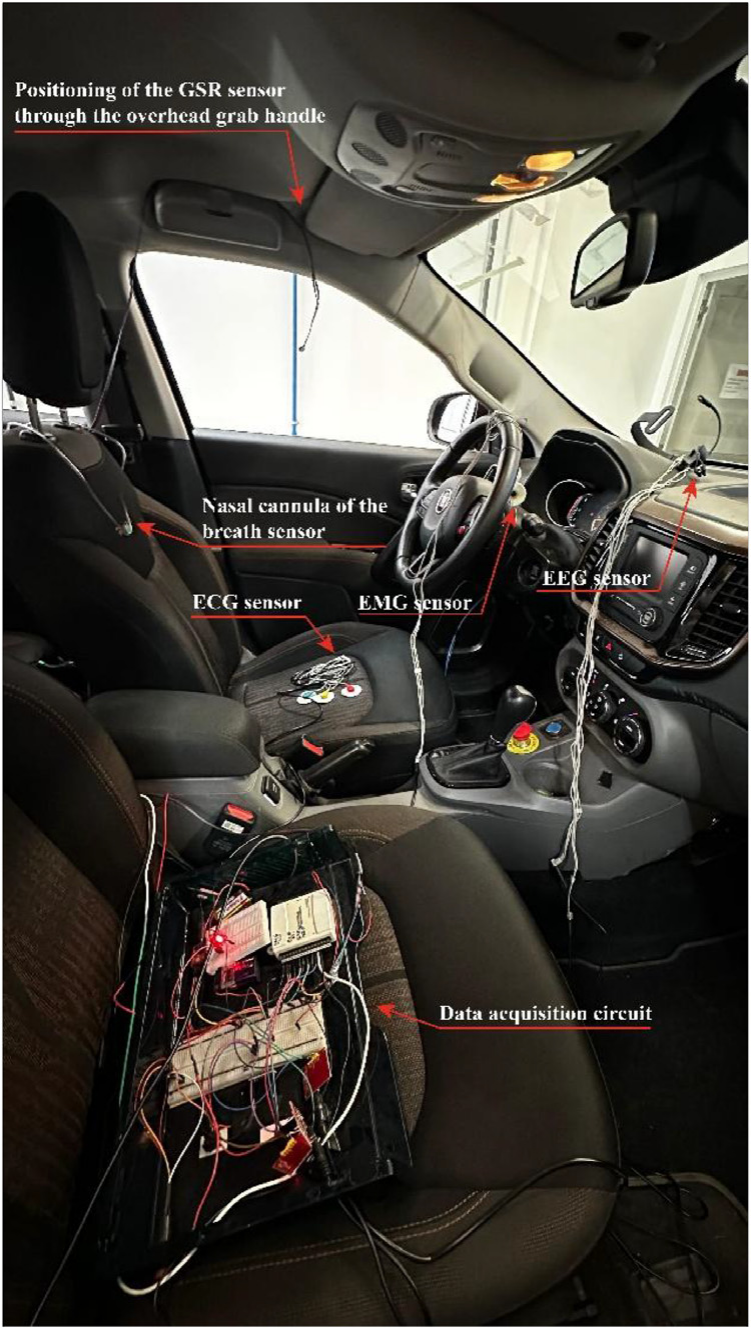
Representation of driver instrumentation inside the vehicle.

Fig. 1 illustrates a screenshot of the LabVIEW program developed for acquiring and storing physiological signals. Dedicated channels and filters were configured for each sensor. Fig. 2 depicts sensors and acquisition boards mounted in the vehicle. An inverter connected to the car battery powered them, ensuring stability during real traffic sessions.

**Fig. 3:**
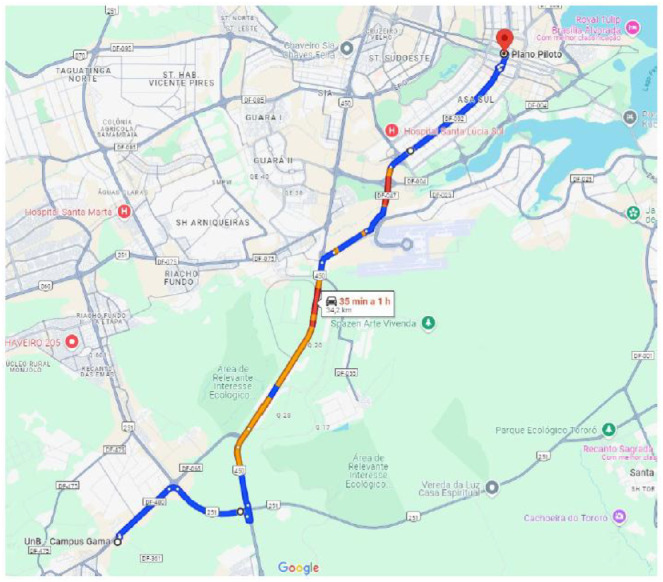
Predefined driving route of 34.2 km between the faculty of science and technology in engineering (FCTE) and the main bus terminal in Brasília (Plano Piloto). Source: Google, 2024.

**Fig. 4:**
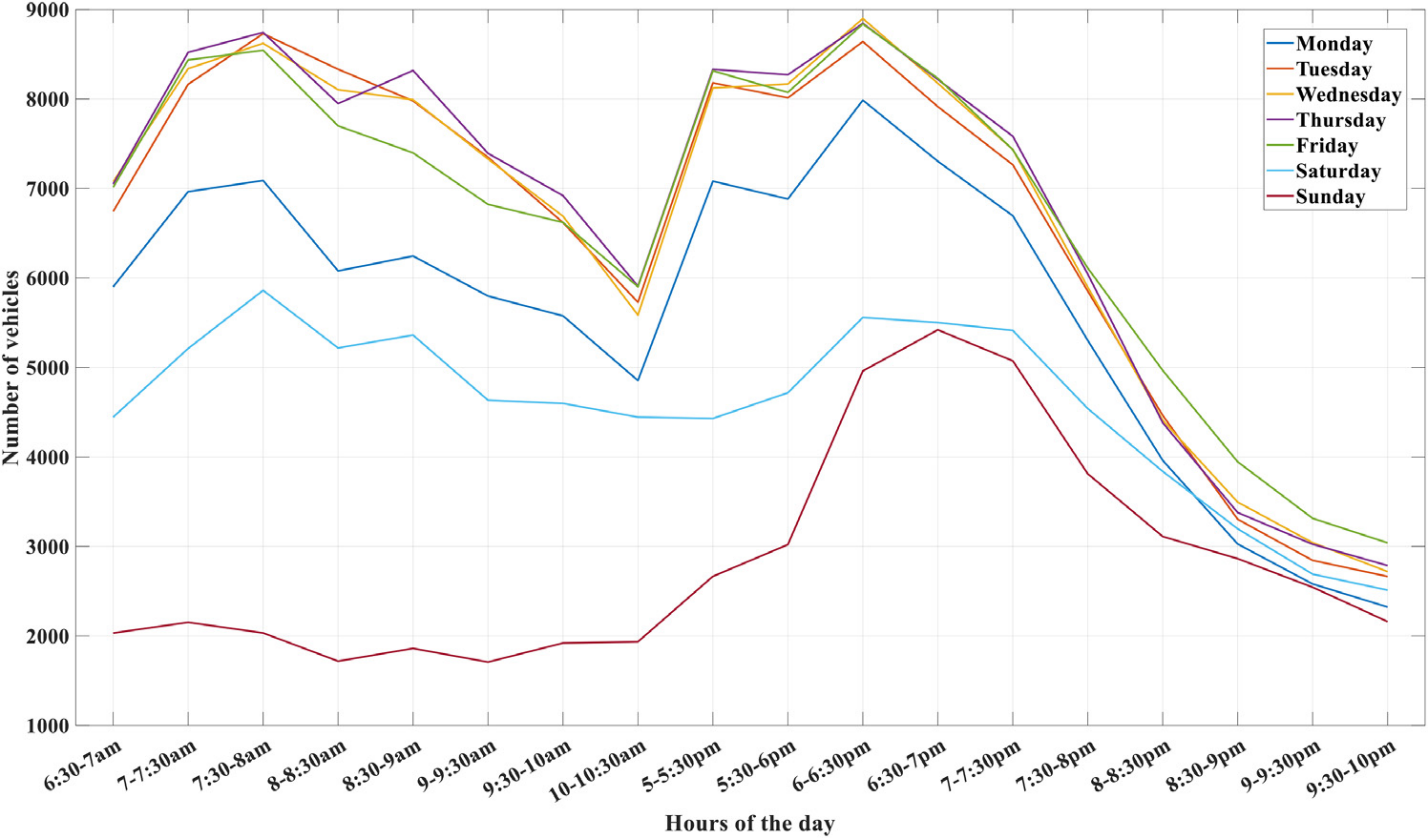
Distribution of traffic flow by weekday and time of day along the selected route. Source: DER-DF.

Driving sessions followed a predefined 34.2 km route between the Faculty of Science and Technology in Engineering (FCTE) and the main bus terminal in Brasília (Plano Piloto), covering urban roads under typical traffic conditions (Fig. 3). Traffic flow characteristics for the selected route were obtained from DER-DF and are included in the repository as contextual data (Fig. 4).

## 4 Experimental Design, Materials and Methods

Seven drivers participated in the on-road data acquisition. The group comprised six male and one female drivers. Participants’ ages ranged from 22 to 62 years (male: 22, 30, 45, 55, 60, and 62 years; female: 28 years). All participants obtained their driver’s licenses at 18 years of age and were active drivers at the time of the study, resulting in driving experience ranging from approximately 4 to 44 years. The participant set was intentionally heterogeneous with respect to age and driving experience; however, analyses aimed at establishing associations between participant profile (e.g., age or years of driving) would require a larger, stratified sample.

The definition of the signal acquisition equipment and protocols was based on the desired characteristics of our dataset and considering the road and traffic conditions in the driveways available for conducting our experiments. In this respect, our main specifications for building the database of signals included the following characteristics.

1. Each acquired signal should reflect real reactions by human drivers, while they operate an automobile in real driving conditions.
2. The driving route should be the same for each driver, for strict signal comparison.
3. The driving route should include regions of intense traffic, so the signals include physiological reactions to stress conditions such as those normally encountered in real driving conditions.
4. The route should require approximately 90 min to 120 min, depending on the driver characteristics and occasional traffic fluctuations. The chosen approximate average duration was aimed at providing sufficient data for our desired correlation analyses, while allowing for driving conditions and drivers emotions and reactions to stabilize.
5. All driver signals should be acquired at a 14-bit depth, and with a sampling frequency of 2 kHz. The quantization level of 14 bits is considered appropriate to all the analysed signals, and the sampling of 2 kHz satisfies the Nyquist criterion in all the cases.
6. Simultaneously to the driver signals, we wanted to acquire information regarding the vehicle conditions, such as accelerations and speeds, by using an on-board diagnostics (OBD) tool.

Regarding the sampling rate (item 5), note that some signals (such as the respiratory signal) present a lower total bandwidth, while others (such as the ECG and the EMG) present higher total bandwidths. So, we could adopt different values for the sampling rate in each case. However, we opted to use a common same sampling frequency, so the synchronization between events and signals, as well as between pairs of signals, could be made based on a single event marker and the acquired sample indices, which are then the same for all driver’s signals.

In this section, we describe the acquisition equipment/software, the preparation of the acqui- sition circuits over the driver, and the experiments’ protocols.

### 4.1 Vehicle and ADAS configuration

Data collection was conducted using a 2023 Jeep Renegade equipped with factory-installed Level 2 Advanced Driver Assistance Systems. The vehicle included the Lane Keeping Assist System (LKAS) integrated within the Lane Sense platform, and the Forward Collision Warning System (FCWS) with automatic braking functionality.

The LKAS operates at approximately 60–180 km/h and provides steering assistance when lane markings are detected. For the experiments, lane departure warning sensitivity was configured to “early” alert mode with high feedback intensity.

The FCWS was configured to “alert + active braking” mode, with the warning distance parameter adjusted to the longest available setting to ensure earlier system activation during vehicle approach events.

All ADAS features were factory-calibrated and manually verified before each session to ensure consistent operation. It is important to note that any firmware alteration has been applied.

### 4.2 Acquisition equipment

The acquisition system was designed to collect five physiological signals: (1) electrocardiogram (ECG), muscle contraction potentials (low-pass filtered EMG), electroencephalography (EMG), galvanic skin response (GSR), and respiration. Each signal was acquired synchronously with respect to the vehicle’s kinematic variables.

Also, each signal was acquired using a dedicated circuit connected to a National Instruments data-acquisition module (the NI USB-6009), interfaced with LabVIEW for Teaching/Research AVL 2024 software, which provided real-time visualization, filtering, and data storage.

The ECG, muscle contraction, EEG, and GSR circuits were based on commercially available devices. Both the ECG and the muscle contraction modules used a custom-built circuit based on the AD8232 chip, with a single analogue acquisition channel. The circuit provides differential amplification and hardware filtering type Bessel order 8, with a 0.5–40 Hz band-pass.

The GSR module, on the other hand, uses a custom circuit based on a voltage divider configu- ration with the Grove GSR sensor (by Seeed Studio). In this module, the GSR signal is measured using silver–chloride electrodes connected to the Grove sensor, which is then interfaced with a signal conditioning circuit to adapt the 0–5 V output range for the NI module input. The circuit measures skin conductance (inverse of resistance), which varies according to the driver’s stress and arousal levels.

Regarding the EEG signal, we used a MindWave Mobile 2 sensor, by NeuroSky Inc., USA. This device measures the EEG waves through a dry electrode placed on the forehead, above the user’s left eye, and an ear-clip reference electrode. The system was selected due to its portability and ease of integration for in-vehicle monitoring of cognitive states such as attention and alertness.

During preliminary testing, however, the wireless interface of the sensor exhibited a factory de- fect that prevented Bluetooth communication between the headset and the acquisition computer. To overcome this issue, the EEG sensor was adapted for wired acquisition. The electrodes from the ear clip and the forehead sensor were connected directly to two AD8232 analogue front-end boards (Analog Devices Inc.), which were subsequently interfaced with the main data acquisition circuit. This configuration enabled simultaneous recording of both EEG channels, forehead and ear reference, under the same synchronized data-acquisition architecture used for the other physiological signals.

The resulting setup allowed the integration of EEG data into the unified signal database, ensuring temporal synchronization and compatibility with the 2 kHz, 14-bit acquisition framework applied across all physiological modalities.

Respiratory activity was monitored using a pressure transducer coupled to a nasal cannula, which measured variations in airflow pressure during inhalation and exhalation. This configuration provided a non-invasive means of quantifying breathing patterns while allowing drivers to perform the tasks naturally within the vehicle environment. The output signal from the pressure transducer was routed to a custom signal-conditioning circuit assembled on two protoboards, each powered by an independent 9 V battery to ensure electrical isolation and reduce interference among channels. The conditioned signal was then digitized through the National Instruments NI USB-6009 data acquisition board, operating at 14-bit resolution and a 2 kHz sampling rate, consistent with the other physiological measurements.

**Fig. 5:**
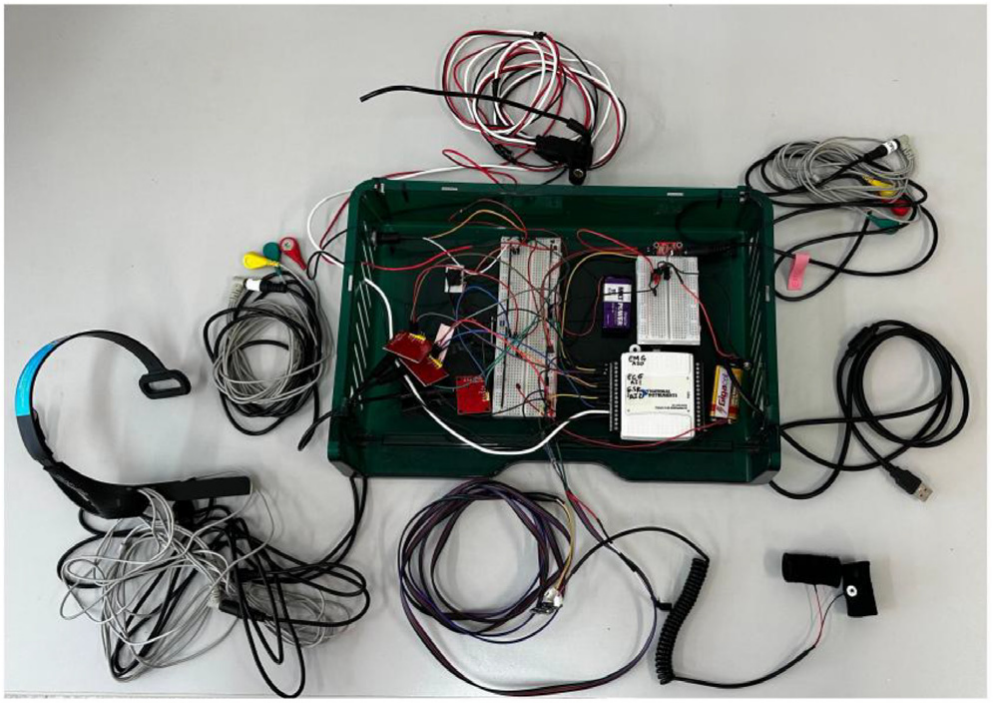
The complete acquisition system, with the sensors for ECG, muscle, respiratory, EEG, and GSR signals. The sensors were connected to a circuit fixed inside an acrylic box.

All electronic components were securely mounted on an acrylic support plate to prevent sensor displacement during vehicle motion, maintaining signal integrity throughout the driving experiments. This configuration proved robust for in-vehicle data collection, enabling stable and synchronized acquisition of respiratory pressure variations across the full duration of each driving session. Fig. 5 presents the final integrated system used for the acquisition of the driver’s physiological signals.

### 4.3 Software and data management

The LabVIEW environment handled multichannel acquisition at 2 kHz per channel, 14-bit resolution, and with timestamp-based synchronization. To improve signal visualization during the acquisitions, we included custom digital lowpass finite impulse response filters type Bessel order 8, with a cutoff frequency of 40 hertz. All the acquired signals, including the filtered versions and the raw, original versions were saved to comma-separated-values (CSV) files.

Vehicle variables, such as velocity, acceleration, and throttle position, were collected using an On-Board Diagnostics (OBD II) interface connected to the CAN bus and integrated to the other acquisition circuits, to ensure a level of temporal alignment.

### 4.4 Preparation of the circuits on each driver’s body

Before each experiment, participants were instructed about the objectives and procedures, and all sensors were installed by the research team to ensure repeatability. The electrodes and sensors were placed according to standardized positions summarized in Table 2.

**Table 2:** Placement of the physiological sensors and description of the corresponding signals, with their purposes in the proposed acquisitions.

Signal	Sensor placement	Purpose / Remarks
ECG	Three disposable Ag/AgCl electrodes: two on the chest and one ground on the lower abdomen	Heart-rate and heart-rate-variability monitoring
EEG	Two frontal electrodes and one reference clip on the earlobe	Brain activity related to attention and mental workload
EMG	Two surface electrodes on the right lower part of the leg, above the foot	Muscle contractions during accelerations and brake activations and control actions
GSR	Two electrodes on the middle and index fingers of the non-dominant hand	Skin conductance linked to stress and arousal responses
Respiration	Pressure sensor over the nose openings	Breathing frequency and pattern monitoring

Electrode sites were cleaned with alcohol to reduce impedance (< 10 kΩ), and cables were routed to minimize motion artifacts. Each participant performed a short calibration stage (3 min) while stationary to verify proper signal quality before starting the driving task.

### 4.5 Experiment’s protocols

Each driver completed a 34.2 km route between the College of Sciences and Technologies in Engineering (FCTE/UnB) and the Brasília central bus station, Fig. 3. The route was selected for its mix of arterial and urban segments, including zones of dense traffic to elicit stress-related physiological responses. This allowed us to observe potential correlations between road conditions (including stressful conditions) and features extracted from the ECG, muscle, EEG, respiratory, and GSR signals.

The experiments began at 7:00 a.m. at the College of Sciences and Technologies in Engineering (FCTE/UnB), during peak morning traffic. They were conducted from Tuesday to Friday aiming to reduce variability associated with weekend traffic patterns. No rainfall occurred during the acquisition days, ensuring comparable environmental conditions across sessions.

Although traffic density corresponded to typical rush-hour conditions and remained broadly consistent, as illustrated in Fig. 4, variations in vehicle flow resulted in moderate differences in average velocity and total travel time among drivers. The mean velocities for the seven participants were 31.1 km/h, 41.0 km/h, 36.0 km/h, 36.0 km/h, 52.6 km/h, 36.0 km/h, and 39.5 km/h. These variations reflect individual driving behaviour and minor differences in traffic flow, while maintaining overall comparable traffic exposure across sessions.

During the drive, the Lane Keeping Assist System (LKAS) and Forward Collision Warning System (FCWS) were active. Our goal was to evaluate whether and how system operation related to variations in physiological signals. Drivers were instructed to operate the vehicle normally and in accordance with standard traffic regulations. The instrumentation was configured to remain as unobtrusive as possible. Researchers were positioned in the rear seat to monitor the acquisition in real time via LabVIEW and to register occurrences of LKAS and FCWS activations based on the visual and auditory alerts provided by the vehicle interface, as well as relevant traffic events. These occurrences were recorded using the acquisition time reference, enabling subsequent alignment with the corresponding physiological and vehicle data segments in the released files.

Because on-road acquisition is affected by natural driver movements and vehicle vibrations, temporary signal dropouts and motion-related artifacts were expected. To curate the released dataset, the research team visually inspected the time series and selected segments in which the acquisition showed no interruption due to loss of electrodes contact. We prioritized continuous intervals suitable for downstream analysis. In addition, researchers recorded the time of relevant driving and ADAS-related events during each session; these timestamps were used as references for segment definition and alignment. For analyses around ADAS activations, time windows centered on each recorded activation were considered using a 30-s pre-event and a 30-s post-event interval. Secondary users should consider that excluded portions may be associated with higher-motion driving moments.

## Limitations

The present work faced some limitations during dataset development. A major challenge was ensuring synchronization between physiological signals and vehicle data. Because acquisition relied on simultaneous recording from different systems, slight delays in transmission or processing could generate inconsistencies in the raw files.

Another limitation concerns the robustness of the instrumentation. Although the acquisition system was functional, further refinements may improve recording stability under complex real-world driving conditions.

The number of participants also limits the dataset, as recordings were obtained from seven drivers under naturalistic traffic conditions. Therefore, the dataset is not intended to provide statistically representative conclusions about the broader driving population. Instead, the present dataset represents a controlled naturalistic resource designed to support methodological investigations and, exploratory analyses involving synchronized physiological and vehicle dynamics data collected during Level 2 ADAS operation. In addition, the inherent noise in physiological signals in traffic environments required careful curation and segmentation procedures, which may introduce selection-related biases that demand attention in secondary analyses.

## Ethics Statement

All participants provided written informed consent through a Informed Consent Form (ICF) prior to participation. The research involved non-invasive physiological monitoring and vehicle data acquisition conducted under normal driving conditions, without altering participants’ routine activities.

Given the observational and non-interventional nature of the procedures, and the absence of clinical or invasive components, the study was not submitted for formal review by a Research Ethics Committee. The procedures were designed to involve minimal risk, and no clinical or interventional procedures were performed. Participant anonymity was preserved through data identification, and no personally identifiable information is included in the released dataset.

The study adhered to ethical principles of voluntary participation, informed consent, risk minimization, and confidentiality consistent with internationally recognized guidelines for research involving human subjects, including the principles outlined in the Declaration of Helsinki.

## Credit Author Statement

**Castro, G. M:** Investigation, Validation; **Oliveira, A. B. de S:** Investigation, Methodology, Reviewing. Miosso; **C. J.:** Investigation, Software, Validation, Writing; **Silva, R. C.:** Conceptualization, Methodology, Original draft preparation, Writing.

## Data Availability

Mendeley DataDataset of physiological signals in the use of advanced driver assistance systems (ADAS) (Original data).
